# Cognitive behavioural therapy for the treatment of depression in people with multiple sclerosis: a systematic review and meta-analysis

**DOI:** 10.1186/1471-244X-14-5

**Published:** 2014-01-09

**Authors:** Daniel Hind, Jack Cotter, Anna Thake, Mike Bradburn, Cindy Cooper, Claire Isaac, Allan House

**Affiliations:** 1 Clinical Trials Research Unit, University of Sheffield, Regent Court, 30 Regent Street, Sheffield S1 4DA, UK; 2 Institute of Brain, Behaviour and Mental Health, The University of Manchester, 3rd Floor, Jean McFarlane Building, Oxford Road, Manchester M13 9PL, UK; 3 Doctorate of Clinical Psychology, Health Research Building, University of Hertfordshire, College Lane Campus, Hatfield AL10 9AB, UK; 4 Clinical Neuropsychology Services, Sheffield Teaching Hospitals, Glossop Road, Sheffield S10 2JF, UK; 5 Leeds Institute of Health Sciences, Charles Thackrah Building, University of Leeds, 101 Clarendon Road, Leeds LS2 9LJ, UK

**Keywords:** Cognitive-behavioural therapy, Depression, Multiple sclerosis, Quality of life, Systematic review

## Abstract

**Background:**

Depression is a common symptom in people with multiple sclerosis. We systematically reviewed published controlled trials on the effectiveness of cognitive behavioural therapy (CBT) for the treatment of depression in people with multiple sclerosis.

**Methods:**

Publications were identified using MEDLINE, PsycINFO and the Cochrane Central Register of Controlled Trials to June/July 2013. We combined thesaurus and free-text terms which were synonyms of the concepts multiple sclerosis, depression and cognitive behavioural therapy. We included published controlled trials which compared individual, group CBT, conducted face-to-face or remotely, to no CBT. Two reviewers extracted data to calculate standardized mean differences (SMD) for self-reported symptoms of depression and weighted mean differences (WMD) for the Multiple Sclerosis Impact Scale (MSIS-29), with 95% Confidence Intervals (CIs). We investigated statistical heterogeneity using I^2^.

**Results:**

Seven eligible studies (n = 433) were identified, which evaluated the effect on depression of CBT delivered individually (3 studies), in a group (3 studies) and by computer (1 study). The summary effect (SMD -0.61, 95% CI -0.96 to -0.26, p=0.0006) was reduced (SMD -0.46, 95% CI -0.75 to -0.17, p=0.002) when an outlying study was removed in a sensitivity analysis to examine statistical heterogeneity. Three studies (n=213) observed a direction of effect using the MSIS-29 which was not statistically significant (WMD -4.36, 95% CI -9.33 to 0.62, p=0.09). There was no between-subgroup heterogeneity (I^2^=0).

**Conclusions:**

CBT can be an effective treatment for depression in MS. Further research should explore optimal durations and modalities of treatment for patients with different characteristics.

## Background

Multiple Sclerosis (MS) is a chronic, immune-mediated condition which affects the central nervous system. Initially, the disease usually involves repeated episodes of symptoms including impaired vision, visual cognition or balance, limb weakness, pain and fatigue. Permanent physical and cognitive disabilities often follow later [1]. Depression is a common co-morbidity in people with MS. Up to 50% of people with MS will experience major depressive disorder in their lifetime [2], a figure considerably higher than that reported for either the general public or many other chronic patient populations [3,4]. Depression in MS has been associated with breakdowns in interpersonal relationships and employment [5], cognitive impairment [6,7], decreased medication adherence [8], heightened suicide risk [9,10] and has been recognised as a major determinant of patient quality of life [11-14]. A variety of aetiologies have been proposed, collectively suggesting a complex interrelationship between biological and psychosocial factors [15-17].

Left untreated, depression in MS does not appear to remit spontaneously [18], however evidence suggests that it is amenable to treatment by both pharmacological and psychotherapeutic interventions [19,20]. Cognitive Behavioural Therapy (CBT) is used to treat depression by conferring skills to identify and reappraise negative thoughts impacting on feelings and behaviours [21]. One focus of CBT has been on the impact of maladaptive coping styles on depression in MS [22]. The use of emotion-focused coping styles has consistently been related to more negative adjustment [23-25] and CBT can be used to support the adoption of more beneficial problem-focused coping styles [17]. CBT techniques can also be applied to reduce perceived disease burden and improve wellbeing [23,26,27].

Previous reviews suggest that CBT for depression has demonstrated promising but inconclusive results in people with MS [28,29]. Three relevant controlled trials have been published since the last systematic review was completed, including the largest study yet conducted on the topic. These trials also use a recently validated disease-specific quality of life instrument, which people with MS report best reflects their concerns [30]. The objective of this review is an up-to-date overview of controlled trials, assessing the impact of CBT on patient-reported measures of depression and disease-specific quality of life in people with MS. Reporting conforms to the PRISMA statement [31]. A PRISMA checklist is provided in Additional file 1.

## Methods

### Protocol and registration

The methods for this review were developed by members of the team and piloted as part of a Masters in Public Health dissertation written in 2008-9 (available on request). In 2009-10, the methods were adapted without drafting or publishing a formal protocol (see Discussion) and the review was completely repeated by the authors. As initial data extraction was completed prior to the inception of the PROSPERO database [32], the protocol was not registered.

### Eligibility criteria

Included studies had populations aged 18 years or over with diagnoses of MS and depression, either as a psychiatric diagnosis or at clinically important levels on a validated depression scale. No restrictions were placed on length of follow-up. Populations with co-morbid dementia or other psychiatric disease unrelated to MS were excluded.

The intervention in eligible studies was CBT delivered by therapists or in a computerised online form. Therapist-led CBT was delivered individually (face-to-face or by telephone) or in groups. Studies evaluating neuropsychological training or cognitive rehabilitation programmes were ineligible. Comparator groups were alternative talking therapies, waiting list controls, ‘standard care’ (however defined) or no treatment for depression. In order to be included, studies had to evaluate self-reported symptoms of depression using at least one validated instrument. Only randomised and quasi-randomised controlled trials were included. Studies not published in English were excluded.

### Literature search

On 10^th^ July 2013 we searched Ovid MEDLINE In-Process & Other Non-Indexed Citations and Ovid MEDLINE 1946 to Present, Ovid PsycINFO 1806 to July Week 1 2013 and the Cochrane Central Register of Controlled Trials: Issue 6 of 12, June 2013 for controlled trials evaluating CBT for the treatment of depression in people with MS. We used terms related to MS, depression and CBT (see Additional file 2 for full search details for each of the databases). We used Google to find home pages of authors of included studies and screened these for references not identified by the searches.

### Study selection

Two researchers (AT and JC) independently screened titles and abstracts for eligibility; differences were resolved by discussion with DH.

### Data extraction

AT and JC used a standardised data extraction form to extract data on the study setting, design, participants and descriptors of the intervention and control group (content and duration of treatments). We abstracted baseline prognostic characteristics, including age, sex, duration and course of disease, MS-specific disability measured by the Expanded Disability Status Scale or the Guy’s Neurological Disability Scale [33,34] and self-reported depression, however measured. We extracted outcome data at baseline and follow-up, as reported in the paper.

### Quality assessment

AT and JC assessed the included studies, unblinded, for generic dimensions of methodological quality associated with estimates of treatment effects in controlled trials: (1) concealment of the allocation schedule; (2) generation of the allocation sequences (randomization); and, (3) inclusion in the analysis of all randomized participants (intention-to-treat analysis), with all clinician withdrawals or patient dropouts accounted for [35]. Disagreement between researchers about any aspect of data abstraction or validity assessment was resolved by recourse to a third reviewer (DH). As the Cochrane Handbook recommends that tests for funnel plot asymmetry should be used only when there are at least 10 studies included in the meta-analysis, we did not evaluate the potential for publication bias [36]. We used the methods described by Dwan and colleagues [37] to assess the presence of Outcome Reporting Bias (ORB). Contact with authors related only to retrieval of unpublished time-points, not outcomes per se. As adequate outcome data on self-reported depression was available for all studies, an ORBIT matrix was not completed. We did not conduct sensitivity analysis to consider robustness to either ORB or study publication bias as the former was not detected, and insufficient studies were retrieved to detect the latter.

### Synthesis

The primary outcome was self-reported symptoms of depression measured on any validated instrument. The secondary outcome was the score on the Multiple Sclerosis Impact Scale (MSIS-29) psychological subscale [38]. We did not pre-specify a time point for assessment of any outcomes, as we were aware of between-study variation in this regard.

As studies assess self-reported symptoms of depression using different scales we pooled estimates of clinical effect using the standardised mean difference (SMD) in which the size of the intervention effect is represented in units of the standard deviations (SD). Negative SMDs indicate differences in self-reported symptoms of depression which favour the CBT arm. Conventionally, values of 0.20, 0.50, and 0.80 indicate, respectively, small, medium, and large effects [39]. Where non-reported outcome data could be neither imputed nor recovered from the authors of the original papers, the effect sizes were deemed not estimable. For MSIS-29 scores, non-standardised differences were pooled; otherwise, the analysis strategy was the same as previously.

The graphs in the results section present the number randomised at all time points where an intention-to-treat analysis was available, but the number analysed at each time-point where an available case analysis was available. Trials were combined using the DerSimonian and Laird random effects model [40]. We used I^2^ to measure the amount of between-study variation in effect estimates which could not be explained by the play of chance alone (statistical heterogeneity). Conventionally, I^2^ values of 25%, 50%, and 75% denote low, moderate, and high levels of inconsistency [41].

In the absence of direct comparisons, we used a test for interaction to test the null hypothesis that there is no difference between individual CBT and group CBT [42].

## Results

### Searches and selection

**Figure 1:**
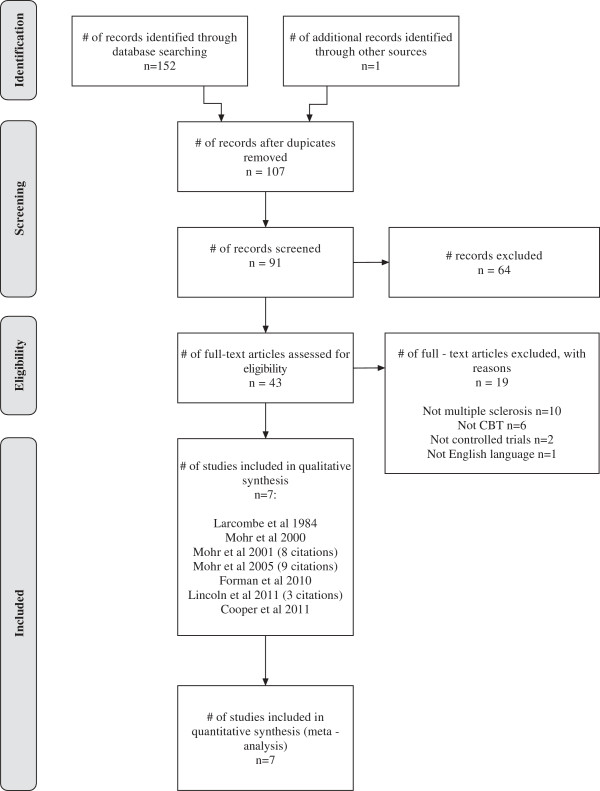
Study flow (PRISMA) diagram.

After eliminating duplicates, 107 unique citations were retrieved from the searches, of which 43 full papers were included. We excluded 19 full papers, because of ineligible populations (n=10), interventions (n=6), language (n=1), and studies not being randomised controlled trials (n=2). Seven discrete studies, represented by 24 citations due to multiple publication, met the inclusion criteria (Figure 1, Table 1).

**Table 1:** Study characteristics

**Study**	**Population**	**Intervention**	**Comparator(s)**	**Outcomes (Primary, Secondary)**
Larcombe (1984) [48]	BDI ≥ 20; Self-reported depression ≥ 3 months; Met Feighner criteria for ‘definite’ or ‘probable’ depression; No psychological co-morbidities; Low suicide risk; Normal memory function; No concurrent or prior treatment with major tranquilisers or lithium. Diagnosis of MS confirmed by a neurologist	Six weekly 90 minute group cognitive behavioural therapy sessions	Waiting list for delayed treatment	BDI; HRSD; Mood Ratings (3 item questionnaire, 10 point scale); Depression as rated by significant other (6 item questionnaire, 4 point scale)
Mohr (2001) [25]	BDI ≥ 16; HRSD ≥ 16; Clinical diagnosis of MDD assessed using SCID; No psychological or neurological co-morbidities, suicidal tendencies or CNS disorders; Willingness to abstain from any other treatment for depression than that provided in the study. Confirmed diagnosis of MS (Poser criteria)	Sixteen weekly 50 minute individually administered cognitive behavioural therapy sessions	Supportive-expressive group therapy; Sertraline	BDI; BDI-18; HRSD; MDD assessed using SCID
Mohr (2005) [47]	BDI-II ≥ 16; HRSD ≥ 14; GNDS ≥ 3 on one or more areas of functioning; No co-morbid dementia, psychosis, substance abuse or suicidal tendencies; Not currently undergoing psychotherapy; No medication other than antidepressants. Diagnosis of MS confirmed by a neurologist	Sixteen weekly 50 minute telephone administered cognitive behavioural therapy sessions	Telephone administered supportive emotion-focused therapy	BDI-II; HRSD; MDD assessed using SCID; Positive affect measured using PANAS-PA
Mohr (2000) [45]	POMS-DS ≥ 15; If in treatment for depression must have been in that treatment for ≥ 3 months; No co-morbid dementia or neurological disorders. Confirmed diagnosis of MS (Poser criteria)	Eight weekly 50 minute telephone administered cognitive behavioural therapy sessions	Standard care	POMS-DS; Post-treatment adherence to IFNβ-1a
Forman (2010) [43]	Diagnosis of MS > 3 months; HADS ≥ 8 or GHQ-12 ≥ 3	Six fortnightly 120 minute group therapy sessions based on cognitive-behavioural and psycho-educational framework	Standard care	HADS; GHQ-12; MSIS; MSSE; SF-36
Lincoln (2011) [44]	Diagnosis of MS > 12 months; HADS ≥ 8 or GHQ-12 ≥ 3. Diagnosis of MS confirmed by a neurologist	Six fortnightly 120 minute group therapy sessions based on cognitive-behavioural and psycho-educational framework	Standard care	BDI-II; HADS; GHQ-12; MSIS; MSSE; EQ-5D
Cooper (2011) [30]	BDI-II ≥ 14 but < 29; EDSS < 8.5; MMSE < 24; No psychological co-morbidities; Low suicide risk; No treatment from psychologist, psychotherapist or psychiatrist within last 3 months. Confirmed diagnosis of MS (McDonald criteria)	Eight 50 minute computerised cognitive behavioural therapy sessions	Standard care	BDI-II; MSIS; SF-36; PHQ-9; GAD-7

BDI: Beck Depression Inventory; EDSS: Expanded Disability Status Scale; GAD-7: Generalised Anxiety Disorder 7-item; GHQ-12: General Health Questionnaire 12-item; GNDS: Guy’s Neurological Disability Scale; HADS: Hospital Anxiety and Depression Scale; HRSD: Hamilton Rating Scale for Depression; MDD: Major Depressive Disorder; MMSE: Mini Mental State Examination; MSIS: Multiple Sclerosis Impact Scale; MSSE: Multiple Sclerosis Self-Efficacy Scale; PANAS-PA: Positive Affect subscale of the Positive and Negative Affect Scale; PHQ-9: Patient Health Questionnaire 9-item; POMS-DS: Profile of Mood States - Depression-Dejection Scale; SCID: Structured Clinical Interview for DSM-IV; SF-36: Short Form Health Survey.

### Study characteristics

The studies were undertaken in the UK (n=3 [30,43,44]), the US (n=3 [45-47]) and Australia (n=1 [48]), and were published between 1984 and 2011. Only three studies reported MS diagnostic criteria, two using the Poser criteria [45,46], one the McDonald criteria [30]. Five studies recruited participants through local MS community centres or outpatient clinics, advertising through MS newsletters and referral from clinical staff specialising in MS [30,43,44,46,48]. Three studies recruited through a medical care program database [30,45,47].

### Population characteristics

The average age of study participants ranged from around 42 [48] to 48 [47] years (Table 2). The average number of years participants had lived with MS varied and was reported inconsistently across studies. Two studies did not report the MS type [47,48]; relapsing remitting was the most common type in other studies with primary progressive disease only represented in two studies [43,44]. All but one study reported the level of MS-related disability: [48] four studies reported Guy’s Neurological Disability Scale averages ranging from about 18 to about 23; two studies reported Expanded Disability Status Scale averages ranging from 2.4 to about 4. In each of five trials [30,44,46-48] who used the BDI, the median scores at baseline were in the moderate range (19-29 points), with two trials [47,48] probably recruiting a significant minority in the more severe range.

**Table 2:** Participant baseline characteristics

**Study**	**Mean age (SD)**	**Sex**	**Mean (SD) MS duration in years**	**MS disease course**	**EDSS/GNDS mean (SD)**	**Depression severity measure mean score (SD)**
Larcombe (1984) [48]	42.5 (NR)	CBT: F7 M2; Ctrl: F6 M4	NR	NR	NR	BDI. CBT: 27.44 (5.64); Ctrl: 29.00 (8.67)
Mohr (2001) [25]	43.9 (10.0)	F46 M17	7.7 (NR)	RR or SP	EDSS: 2.4 (NR)	BDI. CBT: 24.8 (7.1); Ctrl: 23.5 (7.6)
Mohr (2005) [47]	CBT: 48.6 (9.6); Ctrl: 47.3 (10.1).	CBT: F47 M15; Ctrl: F51 M14	CBT: 11.6 (10.1); Ctrl: 10.9 (10.1)	NR	GNDS. CBT: 23.9 (5.8); Ctrl 22.9 (6.7)	BDI. CBT: 27.00 (7.78); Ctrl: 28.32 (7.91)
Mohr (2000) [45]	CBT: 42.6 (12.8); Ctrl: 42.1 (9.4)	CBT: F10 M6; Ctrl: F13 M3	CBT: 6.1 (6.6); Ctrl: 6.1 (6.7)	CBT: RR16; Ctrl: RR16	GNDS. CBT: 19.0 (9.2); Ctrl: 17.9 (9.2)	POMS-DS. CBT: 33.1 (12.4); Ctrl: 27.9 (12.1)
Forman (2010) [43]	CBT: 47.3 (10.3); Ctrl: 47.7 (9.8)	CBT: F16 M4; Ctrl: F16 M4	CBT: 7.3 (5.4); Ctrl: 12.4 (11.4)	CBT: RR13 PP3 SP3 B1; Ctrl: RR13 PP1 SP6	GNDS. CBT: 19.39 (5.55); Ctrl: 25.37 (8.04)	HADS-D. CBT: 9.5 (3.3); Ctrl: 8.5 (4.3)
Lincoln (2011) [44]	CBT: 44.5 (11.1); Ctrl: 47.5 (10.5)	NR	CBT: 9.2 (7.8); Ctrl: 10.5 (8.0)	CBT: RR55 PP4 SP12 B1; Ctrl: RR48 PP11 SP18 B2	GNDS. CBT: 17.3 (7.8); Ctrl: 16.7 (6.9)	BDI. CBT: 23.1 (12.2); Ctrl: 21.9 (8.7)
Cooper (2011) [30]	CBT: 48 (7.7); Ctrl: 42 (7.0)	CBT: F11 M1; Ctrl: F7 M5	NR	CBT: RR7 SP5; Ctrl: RR12	EDSS. CBT: 4.8 (1.7); Ctrl: 3.6 (1.8)	BDI. CBT: 21.0 (4.0); Ctrl: 23.3 (5.2)

B: Benign MS disease course; BDI: Beck Depression Inventory; CBT: Cognitive Behavioural Therapy group; Ctrl: Control group; EDSS: Expanded Disability Status Scale; F: Female; GNDS: Guy’s Neurological Disability Scale; HADS-D: Hospital Anxiety and Depression Scale – Depression subscale; M: Male; NR: Not reported; POMS-DS: Profile of Mood States - Depression-Dejection Scale; PP: Primary-progressive MS disease course; RR: Relapsing-remitting MS disease course; SP: Secondary-progressive MS disease course.

Four trial reports included data about study take-up [30,43,44,47]. A median of 45% (range 12% [30] to 71% [44]) of those approached about the study screened eligible (with the remainder in some studies refusing as well as failing screening). Medians of 18% (range 4% [30] to 49% [44]) of those approached and 50% (range 26% [43] to 85% [47]) of those screening eligible entered the trial.

### Intervention characteristics

In three trials, the intervention was delivered by an individual therapist, face-to-face in one trial [46], and by telephone in two trials [45,47]. Three trials evaluated group therapy programmes, in which patients were randomised into groups consisting of 4-10 individuals [43,44,48]. One trial evaluated computerised CBT (cCBT) [30]. The duration of the intervention ranged from 6-16 weeks (median 8 weeks) with study follow-up period ranging from 4-64 weeks after randomisation or the initiation of treatment. The majority of the studies reported weekly sessions, but two studies evaluated fortnightly therapy given over 12 weeks [43,44]. The duration of therapy sessions ranged from 50-120 (median 50) minutes. CBT interventions typically consisted of efforts to increase engagement with positive behaviours, social interaction, and training to identify and challenge cognitive distortions. Details regarding the composition of individual sessions were generally limited. Five studies stated that a manual was used to guide treatment [43-47]. Patients were provided with a workbook to act as a visual aid in delivering treatment in the two studies that used telephone administered CBT [45,47]. Five studies used CBT interventions that incorporated specific skills for managing common MS related issues including fatigue, pain and stress [43-47]. Additional relaxation techniques were also incorporated in two studies [43,44]. The cCBT intervention consisted of a generic, commercial, interactive, web-based CBT package ‘Beating the Blues’, which had not been adapted to deal with the symptoms of MS [30]. Homework was set in six studies [30,43-45,47,48].

Of the six studies that included contact with a therapist, three used only a single therapist to deliver treatment to all patients randomised to CBT [43,44,48]. Two studies used more than one doctoral level psychologist with between 1-9 years postdoctoral clinical experience [46,47]. Two studies used advanced postgraduate-level researchers with several years’ experience in providing psychotherapy and prior treatment for MS patients [45,48]. Two studies used assistant psychologists to deliver the intervention [43,44]. Therapist treatment fidelity was assessed in two studies using a modified version of the Cognitive Therapy Scale, [46,47] while adherence was informally monitored in one study through review of audio-taped treatment sessions [45]. Three studies did not report attempts to monitor therapist treatment adherence [43,44,48]. In all six therapist-led studies, those delivering the intervention received regular supervision by a senior and experienced clinician. No study reported attempts to monitor the receipt or enactment of CBT techniques by intervention arm participants. In the six studies where data was available, [30,43,45-48] a median of 9% (range 5% [47] to 31% [45]) of those randomised to CBT interventions failed to complete their course.

### Comparator characteristics

Two included studies used ‘active’ comparators, one incorporating a group therapy and a pharmacotherapy arm (which was not used in the meta-analysis below) [46] and one including a telephone-administered psychotherapy comparator arm [47]. Four studies randomised participants to “standard care” control conditions. Of these, two studies restricted access to any other psychotherapeutic interventions for the duration of the trial, [43,45] while two studies did not restrict this treatment in any way [30,44]. One study used a six-week waiting list control [48]. The studies differed in the extent to which they permitted participants to continue with additional therapy outside of that provided in the trial. One study excluded patients who were unwilling to abstain from psychological or pharmacological treatment for depression other than that provided in the study during the treatment period [46]. One study did not permit participants to receive any additional psychotherapy, though 55% of the sample was concurrently using antidepressants [47]. In one of the studies by Mohr and colleagues two patients randomised to CBT received either antidepressant medication or psychotherapy [45]. Cooper and colleagues reported that, within the CBT arm, one patient received additional psychotherapy, while seven patients used antidepressants [30]. The study by Larcombe and Wilson reported one patient in the CBT arm and two participants in the waiting list control received additional antidepressant medication [48]. Two studies did not report additional treatment received by participants during the study period [43,44].

### Quality assessment

The quality of the studies, outlined in Table 3 was variable. Only two studies clearly demonstrated adequate concealment of the allocation schedule [30,44]. Two studies with small sample sizes used block randomisation in such a way that made the randomisation of later blocks predictable thus undermining allocation concealment and opening the study up to selection bias [43,46]. One study reported a quasi-random method of block randomisation generally thought to be inadequate [46]. The median loss-to-follow-up at the time of the primary outcome assessment (made at between 7 and 16 weeks) was 8% (range 4% [47] to 28% [45]); studies that followed up for longer, reported greater attrition at subsequent time-points. Two studies did not perform intention-to-treat analysis, having lost one and two participants to follow-up respectively [43,48]; in each of the other five studies [30,44-47], the use of “last observation carried forward” was necessary to impute continuous data missing at follow-up. Sample sizes ranged from 32 to 122 (median 45) for individual CBT studies and from 20 to 151 (median 38) for group CBT studies. The one cCBT study had a sample size of 24.

**Table 3:** Quality assessment

**First author**	**Allocation concealment**	**Randomisation**	**Blinding**	**Intention to treat (ITT) and withdrawals**	**Attrition at primary outcome timepoint**	**Largest number lost to follow-up**
Larcombe (1984) [48] Australia	Unclear	Unclear	Raters for HRSD were blind to experimental conditions and assessment occasions	1/20 (5%) withdrew (1 CBT). Participant discontinued treatment after one session. No ITT analysis	1/20 (5%) at 7 weeks (1 CBT)	1/20 (5%) did not complete 7 week outcome assessment (1 CBT)
Mohr (2001) [25] USA	Inadequate	Inadequate: Quasi-random, block randomisation	None	11/63 (18%) dropped out of treatment (1 CBT, 4 Group therapy, 6 Sertraline). ITT analysis carried out on all subjects	9/63 (14%) at 16 weeks (3 SEG, 6 Sertraline)	9/63 (14%) did not complete 16 week outcome assessment (3 SEG, 6 Sertraline)
Mohr (2005) [47] USA	Unclear	Unclear: Stratified based on whether patient currently diagnosed as having MDD and using antidepressant medication	All interviewers conducting telephone assessments were blinded to treatment allocation	7/127 (6%) did not complete the 16 weeks of therapy (3 CBT, 4 Control). 6 participants dropped out by their own choice, 1 was removed from the trial due to an irrelevant issue. ITT analysis carried out on all subjects	5/127 (4%) at 16 weeks (2 CBT, 3 Control)	15/127 (12%) did not complete 28 week follow-up (6 CBT, 9 Control)
Mohr (2000) [45] USA	Unclear	Unclear	None	5/32 (16%) dropped out of treatment (5 CBT). CBT: Inability to make phone appointments or reported conflicts with other obligations. ITT analysis carried out on all subjects using last observation carried forward for missing data	9/32 (28%) at 8 weeks (5 CBT, 4 Control). Control: 3 declined final assessment, 1 died (medical problem unrelated to MS)	9/32 (28%) did not complete 8 week outcome assessment (5 CBT, 4 Control)
Forman (2010) [43] UK	Inadequate: Independent researcher held allocation schedule. Small sample made later groups predictable	Adequate: Block randomisation, computer-generated list of random numbers	Single blind. Outcome questionnaires scored and entered onto computer by an independent researcher	7/20 (35%) randomised to group CBT intervention did not attend the group sessions. No ITT analysis	2/40 (5%) at 12 weeks (1 CBT, 1 Control). CBT: Did not return due to bereavement. Control: Did not return due to MS relapse	3/40 (7.5%) did not complete 26 week follow-up (2 CBT, 1 Control)
Lincoln (2011) [44] UK	Adequate: Web-based randomisation system	Adequate: Block randomisation, computer-generated	Data scored and entered onto database by researcher blind to treatment allocation	1/151 subject withdrew (1 Control) shortly after randomisation. ITT analysis carried out on all subjects using last observation carried forward for missing data	20/151 (13%) at 16 weeks (11 CBT, 9 Control). CBT: 2 patients were too ill, 9 failed to return outcome assessment. Control: 1 patient withdrew, 1 was too ill, 7 failed to return outcome assessment	24/151 (16%) did not complete 32 week follow-up (14 CBT, 10 Control)
Cooper (2011) [30] UK	Adequate: Web-based randomisation system	Adequate: Computer-generated	Statisticians and PI remained blind to treatment allocation codes until after the final analysis	1/12 (8%) randomised to computerised CBT formally requested discontinuation of treatment citing time and lack of enthusiasm as reasons. ITT analysis carried out on all subjects using last observation carried forward for missing data	3/24 (12.5%) at 8 weeks (3 CBT)	6/24 (25%) did not complete 21 week follow-up (2 CBT, 4 Control)

The risk of ORB was deemed low. No study was excluded because authors failed to use a validated depression inventory (primary outcome). Included studies which did not use the MSIS-29 all commenced prior to published work on its validation [38,49].

### Synthesis

**Figure 2:**
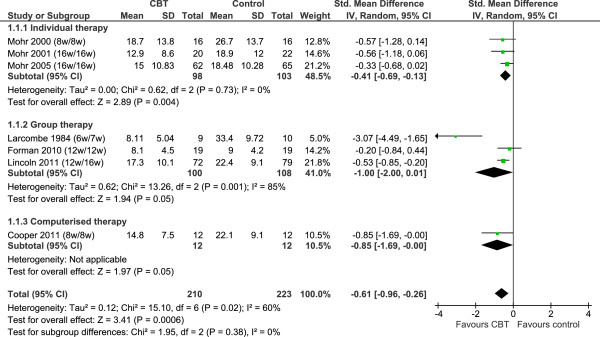
**Depression: post-treatment scores.** Note: All forest plots used display available case analyses for the Larcombe and Forman studies, but intention-to-treat analyses for other studies. See Table 3 for numbers lost to follow up at the post-treatment assessment.

**Figure 3:**
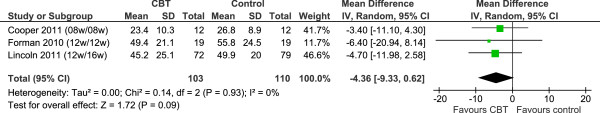
MSIS psychological subscale: post-treatment scores.

**Figure 4:**
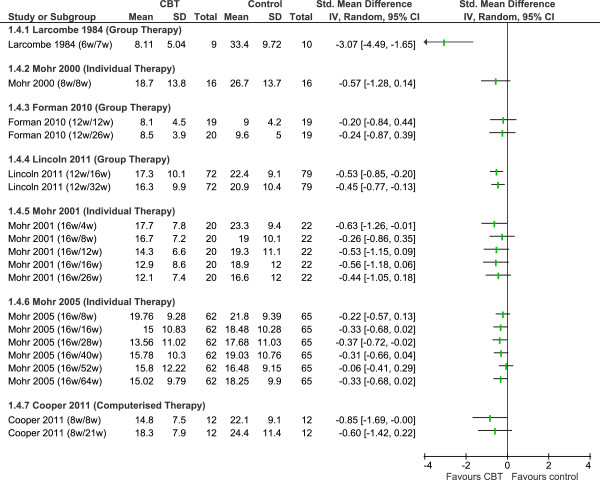
Depression: long-term follow-up assessments.

**Figure 5:**
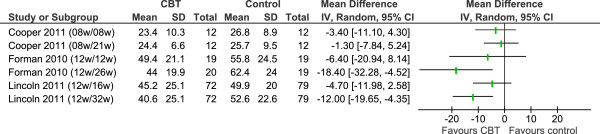
MSIS psychological subscale: long-term follow-up assessments.

We used Review Manager 5.1 to meta-analyse study outcomes. In the forest plots (Figures 2, 3, 4 and 5), the first author and publication date is followed by the duration of therapy in weeks and the timing of follow-up expressed as the number of weeks since randomisation. The Larcombe and Forman teams presented available case analyses, and the denominators for these studies in graphs express the number analysed, rather than the number randomised [43,48]. All 7 studies reported mean (SD) post-scores.

Seven studies (n = 433) compared between 6 and 16 weeks of individual, group or computerised CBT to no CBT (Figure 2). Studies employed summary scores from different inventories measuring symptoms of self-reported depression: BDI; [46,48] BDI-II; [30,44,47] POMS-DS; [45] HADS-D [43]. At end of treatment, CBT of any type and duration conferred an average difference in self-reported symptoms of depression of 0.6 SD (SMD -0.61, 95% CI -0.96 to -0.26, p=0.0006). High levels of statistical heterogeneity (I^2^ = 60%) suggest this result should be interpreted with caution, but this appeared to be driven by a single ‘outlier’ study [48]. When this study was removed in a *post hoc* sensitivity analysis, all between-study variation could be explained by the play of chance alone (I^2^ = 0%) and the clinical effect size was reduced (SMD -0.46, 95% CI -0.75 to -0.17, p=0.002). There was no between-subgroup heterogeneity for the main analysis (I^2^ = 0%), with or without this study included.

In the individual therapy subgroup, three studies (n = 201) compared between 8 and 16 weeks of CBT to no CBT. At end of treatment, CBT conferred an average improvement in self-reported symptoms of depression of 0.4 SD (SMD -0.41, 95% CI -0.69 to -0.13). There was no statistical heterogeneity within this subgroup.

In the group therapy subgroup, three studies (n = 208) compared between 6 and 12 weeks of CBT to no CBT. At end of treatment, CBT conferred an average improvement in self-reported symptoms of depression of 1.0 SD (SMD -1.00, 95% CI -2.00 to 0.01). There were high levels of heterogeneity (I^2^ =85%); removal of the Larcombe study from the analysis [48], discussed above, eliminated statistical heterogeneity in the subgroup, but reduced the clinical effect size to 0.5 SD (SMD -0.46, 95% -0.75 to -0.17). Possible interpretations of the observed statistical heterogeneity are discussed below.

The one remaining study (n=24) investigated 8 weeks of cCBT to standard care, reporting a difference of 0.8 SD (SMD -0.85, 95% CI -1.69 to -0.00).

An indirect comparison of individual vs. group CBT was undertaken by comparing the pooled mean differences for individual therapy and group therapy. The comparison gave a standardised mean difference of 0.59 (95% CI: -0.45 to 1.63; p=0.27) when including all 6 studies, or 0.05 (95% CI: -0.35 to 0.45; p=0.81) when removing the outlying study. We conclude that there is not enough evidence to suggest a difference exists between these individual and group CBT for the treatment of depression in people with MS.

Summary scores at end of treatment were available for the MSIS psychological subscale from three studies (n=213, Figure 3), which compared between 8 and 12 weeks of CBT to no CBT. At end of treatment, CBT conferred an advantage in self-reported MS-specific psychological symptoms of 4 points (WMD -4.36, 95% CI -9.33 to 0.62, p=0.09). This effect translates to a change of 0.23 standard deviations (SMD -0.23, 95% CI -0.50 to 0.04, p=0.09). There was no statistical heterogeneity in either of these analyses (I^2^ =0).

Due to the clinical heterogeneity of programme content, duration and follow-up times we did not consider it appropriate to undertake a statistical synthesis of a longer follow-up period for either self-reported symptoms of depression or the MSIS psychological subscale. The outcome assessments made at various follow-up times within the individual studies are presented in Figures 4 and 5.

## Discussion

### Principal findings

When treating depression in people with multiple sclerosis, CBT appears to confer a medium treatment effect (0.5 SD) compared with standard care and some alternative psychotherapeutic interventions. In the small number of studies where data was provided, CBT also improved disease-specific quality of life in comparison to standard care.

### Study limitations

The methods for this review were based those developed by the team and used in a Masters dissertation (available on request); the research question and inclusion criteria were established before the conduct of the review – a criterion of the AMSTAR tool, used to assess the quality of systematic reviews [50]. Initial data extraction was completed before the development of the PROSPERO database [32]. As the Cochrane Handbook notes, early publication of a protocol for a review prior to knowledge of the available studies reduces the impact of review authors’ biases and allows peer review of the planned methods [36]. Two departures from the original methods did occur in this review. The assessment of outcome reporting bias was undertaken because became best practice, while the review was ongoing [51]. The use of CINAHL, EMBASE and grey literature searching were also abandoned, due to resource constraints and because none had produced unique references in the pilot work. Searching Medline and specialised databases only, along with checking reference lists and contacting experts, can be justified under resource constraints in some topic areas [52]. MEDLINE identifies 97% of all RCTs of cognitive therapy for depression [53], whereas CINAHL in particular, rarely retrieves unique references for most topic areas [54]. For these reasons, we believe the risk of bias associated with our search strategy is low.

Moderate to high levels of statistical heterogeneity, together with the low number of studies and the risk of bias resulting from participant attrition mean these results should be interpreted with caution. Statistical heterogeneity in the meta-analysis indicates that there are underlying clinical and methodological differences (variations in populations, interventions and outcome assessments) between the studies. Much of the heterogeneity could be attributed to a single small study which reported a very large effect [48]. Formal meta-regression was not undertaken but several differences between the Larcombe study [48] and the other group therapy studies may explain the disparity in effect sizes. The Larcombe study [48], conducted in Australia was published twenty-five years prior to Forman [43] or Lincoln [44], both conducted in the UK. Larcombe [48] had higher mean baseline scores on the BDI than the large Lincoln study [44] (Table 2; no comparable data for Forman [43]). There was proportionately higher levels of dropout in the large Lincoln trial (n=151) [44], which provided the majority of the weight for this subgroup, than the smaller Larcombe trial (n=20) [48] (Figure 2; Table 3). Clinical effect sizes in psychotherapy trials tend to be significantly smaller in studies with a sample size of greater than 50 or intention-to-treat analyses (Lincoln [44]) than in smaller studies or those with available case analyses (Larcombe [48] and Forman [43,55]). This aside, the results are arguably more consistent than may have been expected from such diverse settings and protocols. Nevertheless, with so few studies the estimate of heterogeneity will necessarily be imprecise; for instance, although individual CBT versus control had an estimated I^2^ of zero, its upper 95% confidence limit was 77%. Exploration of the impact of factors on effect sizes via meta-regression was not undertaken due to the multitude of potential explanatory factors, limited data and a lack of standardisation in reporting of primary research studies.

The pre-specified ineligibility of non-English language studies resulted in the exclusion of one Persian language study at the full paper stage. As far as we can tell from the English abstract, this paper was not assessing self-reported depression using a validated inventory, focusing rather on a concept described as “hope of life”. It is unlikely the exclusion of this study increases the risk of bias in our own: there is no evidence that the use of language restrictions in systematic review-based meta-analyses causes systematic bias, although it can reduce the precision of pooled estimates [56].

### Key messages for patients, therapists, policy-makers

CBT may provide modest benefits in the treatment of depression in some people with MS and can be provided alongside or as an alternative to pharmacological interventions. The meta-analysis suggests that delivery modes other than traditional, one-to-one, face-to-face, therapist-led CBT may be effective but, although remote delivery can provide greater patient accessibility it also presents certain challenges. In telephone-administration, the lack of visual cues and non-verbal communication require additional paperwork and experienced clinicians to facilitate interaction effectively [45]. Patients have reported finding cCBT physically burdensome, insufficiently supported for proper application of the CBT model and exacerbating feelings of social isolation [57].

Where reporting was adequate, the review highlighted poor consent rates in some trials, which may reflect the perceived attractiveness of CBT interventions as well as of the research protocol. Whilst adherence to treatment was generally good (higher than 90% completion) drop out from one group [43] and one telephone CBT [45] intervention exceeded the average dropout from psychotherapy of 22% cited elsewhere [58] by some margin. Where researchers provided an explanation for poor take up or adherence, it focused on patients prioritising therapy over other commitments at the available times [43,45]. Whilst this could be framed in terms of resource-limited services failing to offer flexibility and choice, it could also indicate a low value placed on talking therapies by the target client group.

Levels of self-reported depression were relatively low in the trial populations, with all averaging within the moderate range (20-28) on the BDI. Researchers who consider the use of somatic symptoms common to MS in depression inventories as potentially flawed may also consider the burden of affective symptoms reported by the trial populations to be exaggerated [59,60]. This is relevant because the response to medication in those with moderate depression is often quite poor. A meta-analysis of data submitted to the Food and Drug Administration found “virtually no difference” in treatment effect between people with moderate levels of initial depression receiving antidepressants or placebo [61]. It is also important to note that the majority of patients included in these trials had been diagnosed with MS for many years. The causes of depression at this time are likely to differ from those in the early years following diagnosis when patients are at the greatest risk of suicide [62,63].

The effect size of 0.5 SD, which equates to between 3-4 points on the BDI, is similar to that observed at comparable follow-up times in a Cochrane review of trials comparing antidepressants with placebo for the treatment of depression in physically ill people [64]. Modest treatment effects, together with the comparably high costs of staffing a clinical service may mean that bespoke CBT interventions for people with MS may not be considered cost-effective at conventional thresholds. One economic analysis, which identified a 38% chance of relapse with psychotherapy compared to 55% chance with pharmacotherapy alone, was unable to conclude face-to-face, therapist-led CBT would be cost-effective in those with moderate depression [65]. The growing availability in some countries of low-intensity telephone CBT [66] and the drive to manage a range of MS symptoms, including fatigue, through nurse-led group CBT interventions [67,68] may mean that services are available for those who wish to access them.

### Need for further research

There are a number of methodological limitations in the current studies that should be addressed in future research. The studies in our meta-analysis are small (mean n=62) compared to those in other meta-analyses on depression in physically ill people. The external validity of the studies is likely to be limited, with only a small number of therapists delivering the intervention in each. To improve the generalisability of findings, larger multi-centre trials are needed to ensure adequate patient recruitment in addition to the use of a wider number of therapists. Future trials must include follow-up periods of at least one year to examine the longer-term effectiveness of CBT interventions, along with the potential for further booster sessions if necessary. Studies should be designed with active comparators, such as supported self-management or a variant of behavioural activation.

More studies incorporating drug comparator arms are required. Only one of the studies in this review compared the use of CBT to a pharmacological intervention [46]. The result that antidepressant medication and CBT show comparable levels of effectiveness in mild to moderately depressed individuals is not exclusive to MS patients [58]. Systematic reviews suggest that the combination of CBT and antidepressant medication may be the most effective mode of treatment for depression, without taking concomitant physical illness into account [69,70]. While the Goldman Consensus statement on depression in multiple sclerosis also favours combination therapy [20], their recommendation is not currently underpinned by controlled trials involving people with multiple sclerosis.

There is a need for greater emphasis on disease-specific quality of life outcome measures to be recorded in conjunction with measures of depression severity. These provide an indicator of how depression and disability impact on the daily lives of people with MS more effectively than self-reported depression instrument scores alone. The MSIS in particular has received support as a useful patient-reported outcome measure from patients, clinicians and researchers [30,57,71].

A large proportion of patients showed no improvement in depressive symptoms, therefore a potentially important direction for further research could be to establish what individual characteristics or physical disease markers signal that a patient may be particularly suited to a particular treatment such as CBT. Some work in this area has begun to emerge, identifying baseline factors associated with treatment response including patient social support [72] and measures of neuropsychological functioning [73]. No study has yet evaluated either the cost-effectiveness of different durations or intensities of CBT programmes. One study has compared the cost-effectiveness of group CBT to standard care, concluding that there were statistically significant differences in the average costs of care favouring CBT [74]. The authors presented incremental cost-effectiveness ratios using cost per point reduction on the BDI-II (£118), rather than employing quality-adjusted life years, favoured by health care commissioners. Future research needs to account for the varying needs of different patients and investigate whether a tailored approach to CBT delivery is possible, which balances value for money with patient acceptability.

## Conclusions

CBT can be an effective intervention for reducing moderate depression, over the short-term, in patients with MS. Early evidence suggests this may also improve patient quality of life. Further research is needed to evaluate longer-term maintenance and the cost-effectiveness of this intervention within this population.

## Competing interests

DH and CC were trialists in the CBT Software for the treatment of depression in people with MS (CoSMoS) trial (Cooper 2011).

## Authors’ contributions

DH was the lead investigator, conceived of the study, designed the searches and, with JC, drafted the manuscript. DH and MB designed the study. DH, JC and AT ran searches, screened studies for eligibility, extracted data and critically appraised primary research studies. DH, JC and AT entered data into Review Manager and undertook meta-analysis. MB was the study statistician and contributed to the first and subsequent drafts of the manuscript. CI and AH contributed psychological and neuropsychological perspectives. DH, JC, AT, MB, CC, CI and AH read, commented on and approved the final manuscript.

## Pre-publication history

The pre-publication history for this paper can be accessed here:

http://www.biomedcentral.com/1471-244X/14/5/prepub

## Supplementary Material

Additional file 1 PRISMA 2009 Checklist. Click here for file

Additional file 2 Search strategies. Click here for file
