# Supplementary material for: Cognitive behavioural therapy for the treatment of depression in people with multiple sclerosis: a systematic review and meta-analysis
Source: BMC Psychiatry. 2014 Jan 9;14:5. doi: 10.1186/1471-244X-14-5 (PMC3890565; doi:10.1186/1471-244X-14-5)
Supplement: Additional file 2 — Search strategies. [file 1471-244X-14-5-S2.pdf]

MEDLINE Search Strategy

1. exp multiple sclerosis/
2. exp myelitis, transverse/
3. exp neuromyelitis optica/
4. exp demyelinating diseases/
5. exp optic neuritis/
6. disseminated sclerosis.mp.
7. ms.mp.
8. or/1-7
9. exp depression/
10. exp depressive disorder, major/
11. exp depressive disorder/
12. exp anxiety/
13. exp psychiatric status rating scales/
14. depress$.tw.
15. dysthymia.mp.
16. anxiety.mp.
17. or/9-16
18. exp cognitive therapy/
19. exp behavior therapy/
20. cognitive behavioural therapy.mp.
21. or/18-20
22. 8 and 17 and 21

PsycINFO Search Strategy

1. exp multiple sclerosis/
2. disseminated sclerosis.mp.
3. ms.mp.
4. or/1-3
5. exp depression/
6. exp anxiety/
7. exp dysthymic disorder/
8. depress$.tw.
9. anxiety.mp.
10. dysthymia.mp.
11. exp quality of life/
12. or/5-11
13. exp cognitive therapy/
14. exp behavior therapy/
15. cognitive behavioural therapy.mp.
16. or/13-15
17. 4 and 12 and 16

Cochrane Central Register of Controlled Trials Search Strategy

1. cognitive behavi*
2. multiple sclerosis
3. 1 and 2
